# Factors associated with delays in pulmonary tuberculosis diagnosis and treatment initiation in Cali, Colombia

**DOI:** 10.26633/RPSP.2019.14

**Published:** 2019-03-15

**Authors:** Cindy Córdoba, Lucy Luna, Diana M. Triana, Freddy Perez, Lucelly López

**Affiliations:** 1 Secretaría de Salud Pública Secretaría de Salud Pública CaliValle Colombia Secretaría de Salud Pública, Cali, Valle, Colombia.; 2 Secretaría de Salud Pública del Meta Secretaría de Salud Pública del Meta VillavicencioMeta Colombia Secretaría de Salud Pública del Meta, Villavicencio, Meta, Colombia.; 3 Pan American Health Organization Pan American Health Organization Communicable Diseases and Environmental Determinants of Health Department Washington, D.C. United States of America Communicable Diseases and Environmental Determinants of Health Department, Pan American Health Organization, Washington, D.C., United States of America.; 4 Universidad Pontificia Bolivariana Universidad Pontificia Bolivariana MedellínAntioquia Colombia Universidad Pontificia Bolivariana, Medellín, Antioquia, Colombia.

**Keywords:** Tuberculosis, pulmonary, diagnosis, therapeutics, delayed diagnosis, Colombia, Tuberculosis pulmonar, diagnóstico, terapéutica, diagnóstico tardío, Colombia, Tuberculose pulmonar, diagnóstico, terapêutica, diagnóstico tardio, Colômbia

## Abstract

**Objective.:**

To determine factors associated with delays in pulmonary tuberculosis diagnosis and treatment initiation in the city of Cali, Colombia.

**Methods.:**

This was a retrospective cohort study of cases of tuberculosis (TB) reported in the TB control program of Cali between January and December 2016. The information was collected from the databases of the TB control program, individual treatment cards, and clinical histories. The variables considered were sociodemographic factors, clinical factors, substance use, and performance of the health service.

**Results.:**

A total of 623 cases were identified, of which 57.0% were male. The median age was 42 years (interquartile range (IQR): 27–60). The median time from onset of symptoms to TB diagnosis was 57 days (IQR: 21–117), and from onset of symptoms to TB treatment initiation was 72 days (IQR: 35–145). A factor associated with longer time from the onset of symptoms to TB treatment was being a previously treated TB patient (coefficient: 123.8 days, 95% confidence interval (CI): 48.3 to 199.3). In contrast, being incarcerated was a protective factor for earlier TB treatment initiation (coefficient: -57.3 days; 95% CI: -92.4 to -22.3).

**Conclusions.:**

Our results provide important information concerning risk factors that are associated with delays in the diagnosis and treatment of tuberculosis, and that are subject to future interventions. Health insurance program managers must work together with health care providers on issues that include patient care, health promotion, and updating TB protocols and standards.

## RESUMEN

**Objetivo.:**

Determinar los factores relacionados con los retrasos en el diagnóstico e inicio del tratamiento de la tuberculosis pulmonar en la ciudad de Cali (Colombia).

**Métodos.:**

Este fue un estudio de cohortes retrospectivo de los casos de tuberculosis notificados en el programa de control de la tuberculosis de Cali entre enero y diciembre del 2016. La información se recopiló a partir de las bases de datos del programa de control de la tuberculosis, las tarjetas de tratamiento individuales y las historias clínicas. Las variables consideradas fueron factores sociodemográficos, factores clínicos, consumo de tabaco y alcohol y funcionamiento del servicio de salud.

**Resultados.:**

Se identificaron 623 casos, 57,0% de los cuales eran hombres. La mediana de la edad fue de 42 años (recorrido intercuartílico [RIC]: 20-60). La mediana del lapso entre el inicio de los síntomas de tuberculosis y el diagnóstico fue de 57 días (RIC: 21-117), y entre el inicio de los síntomas y el inicio del tratamiento antituberculoso, de 72 días (RIC: 35-145). Un factor relacionado con un lapso más largo entre el inicio de los síntomas y el inicio del tratamiento antituberculoso fue el ser un paciente con tuberculosis que ya había recibido tratamiento (coeficiente: 123,8 días; intervalo de confianza [IC] de 95%: 48.3 a 199,3). Por el contrario, estar en prisión fue un factor protector para un inicio más temprano del tratamiento antituberculoso (coeficiente: -57,3 días; IC de 95%: -92.4 a -22,3).

**Conclusiones.:**

Nuestros resultados brindan información importante acerca de los factores de riesgo re-lacionados con los retrasos en el diagnóstico y el tratamiento de la tuberculosis, que son susceptibles de intervenciones futuras. Los directores de los programas de seguro de enfermedad deben colaborar con los prestadores de atención sanitaria en aspectos tales como la atención de los pacientes, la promoción de la salud y la actualización de los protocolos y normas relativos a la tuberculosis.

## RESUMO

**Objetivo.:**

Determinar os fatores associados à demora no diagnóstico e no início do tratamento da tuberculose pulmonar na cidade de Cáli, Colômbia.

**Métodos.:**

Estudo retrospectivo de coorte de casos de tuberculose notificados como parte do programa de controle de tuberculose de Cáli entre janeiro e dezembro de 2016. Os dados foram coletados das bases de dados do programa de controle de tuberculose, fichas individuais de tratamento e histórias clínicas. As variáveis consideradas foram fatores sociodemográficos, fatores clínicos, uso de substâncias químicas e desempenho do serviço de saúde.

**Resultados.:**

Ao todo, foram identificados 623 casos, sendo 57,0% no sexo masculino. A média de idade foi 42 anos (intervalo interquartil [IIQ] 27-60). A mediana de tempo do início dos sintomas ao diagnóstico da tuberculose foi de 57 dias (IIQ 21-117) e do início dos sintomas ao início do tratamento da tuberculose foi de 72 dias (IIQ 35-145). Ter sido tratado anteriormente para tuberculose foi um fator associado a um intervalo maior entre o início dos sintomas e o tratamento da tuberculose (coeficiente 123,8 dias, intervalo de confiança de 95% [IC 95%] 48,3 a 199,3). Por outro lado, estar em detenção foi um fator de proteção para o início precoce do tratamento da tuberculose (coeficiente –57,3 dias; IC 95% –92,4 a –22,3).

**Conclusões.:**

Os nossos resultados contribuem com aporte importante sobre os fatores de risco associados à demora no diagnóstico e no tratamento da tuberculose e são passíveis de intervenções futuras. Os responsáveis pelos programas dos planos de saúde devem colaborar com os profissionais da saúde na assistência ao paciente, promoção da saúde e atualização dos protocolos e padrões de atenção da tuberculose.

Tuberculosis (TB) continues to be a serious public health problem despite global, national, and local strategies for its elimination. The World Health Organization (WHO) stated in its *Global Tuberculosis Report 2017* that during 2016 there were 10.4 million new cases of TB and that 1.68 million people died from this disease (1). In 2016, Colombia reported a total of 12 581 cases of TB. However, it is estimated that 3 000 people with the disease had not been diagnosed (2). In the city of Cali, Colombia, from January through December 2016, 106 deaths due to tuberculosis were reported, making up 10% of the total number of deaths in the city that year (3).

This mortality from TB could be related to a late diagnosis due to the lack of identification of people with respiratory symptoms, an inability of the health system to provide treatment once the patient is identified, and barriers to adherence to treatment (4). In Colombia, according to the national monitoring and evaluation guidelines, the period from the onset of symptoms to an early TB diagnosis should be less than 30 days (5). For the country overall, delays in TB diagnosis have been associated with patients being unemployed or internally displaced; with not having any health insurance; and with not having a contributive type of health insurance that is available to persons with a greater capacity to pay for health insurance (6).

In Brazil, a delay in seeking care has been shown to be associated with being a male between 18 and 29 years old (7). In Ethiopia, the delays in the diagnosis were not significantly different according to sex, but there was a greater topological delay among men, and patients with less education were more likely to present delays (8).

The city of Cali has 2 420 000 inhabitants and is located in southern Colombia. The city has a tuberculosis program that follows the national program standards, which are based on WHO recommendations. The program is implemented in both public and private less-complex health care institutions that receive periodic technical assistance and monitoring from the Cali TB control program, which in turn receives technical assistance from the National Institute of Health (INS) and the Ministry of Health in all that corresponds to TB guideline protocols and regulations.

In the early 1990s, Colombia launched a major health sector reform, which established a health insurance system with two main regimens: a subsidized one and one with contributive health care policies. Participants in the subsidized regimen represent the poorest and most vulnerable population, who are unable to pay the full health costs and thus require a total or partial subsidy to be able to receive health care services (9). Insurance under the contributive health care regime in Colombia is mandatory for people with an employment contract, pensioners or retirees, and self-employed persons with the capacity to pay to cover themselves and their family members (10).

In Cali, 15 to 20 cases of drug-resistant TB are diagnosed annually. The city is classified as a high-risk location for TB due to having a TB incidence level that is twice that of the nation overall. Approximately 90 to 100 people die each year from TB in Cali (3).

In 2015, health authorities in Cali pointed out to health care institutions and the community at large the delay in TB diagnosis. Those officials also established the goal that 80% of pulmonary TB cases would have an early diagnosis. However, the percentage of early TB diagnosis ranges between 60.0% and 67.0% (2). The objective of this study was to determine the risk factors associated with the delay in TB diagnosis and TB treatment initiation in pulmonary TB patients in Cali during the year 2016.

## MATERIALS AND METHODS

### Study design and setting

We conducted a retrospective cohort study using records from Cali’s TB control program from January through December 2016. For inclusion, individuals had to be more than one year old and have a diagnosis of pulmonary TB. In this study, pulmonary TB was identified as any patient that met the clinical or bacteriological diagnostic criteria. For clinical criteria, the patient had to present a positive clinical chart and positive radiology. The bacteriological criterion took into account the clinical chart and a positive sputum smear. Other criteria required for inclusion were being a resident of the urban area of Cali and the availability for this study of at least 80% of the study variables. Patients diagnosed with extrapulmonary TB or who had inconsistencies in their data or duplicate records were excluded.

### Data collection

Information was collected from the databases of the Cali TB control program, individual treatment cards, and clinical charts. Data was entered into Microsoft Excel software with double entry to guarantee the quality of data, and then exported to Epi Info 7.1 software.

The outcome variables were the number of days from the onset of symptoms until TB diagnosis, and from TB diagnosis to TB treatment. Delay in TB diagnosis was defined as ≥ 30 days from the onset of respiratory symptoms to bacteriological confirmation of TB or radiological diagnosis. The variables considered were: 1) sociodemographic factors: sex, age, ethnic group (indigenous, mestizo, or Afro-descendant), health care workers, population groups with vulnerable conditions (homeless or displaced people, prisoners, persons with disabilities, pregnant women), type of health insurance affiliation; 2) clinical factors: coinfection with HIV, diabetes, and malnutrition; and 3) substance use (smoking and alcoholism). The health care service institution rating was also used. The rating was defined as the percentage of compliance with TB regulations, which was estimated during the rating technical assistance and included tuberculosis, information systems, and health care personnel. The rating ranged from 0% to 100%, classified as: 1) low adherence (less than 70%), 2) moderate adherence (70% to 80%), and 3) good adherence (> 80%). In this study, pulmonary TB was defined as any patient admitted to the program as new, or previously treated, with a sputum smear-negative result, a positive culture, and a GeneXpert molecular test (GeneXpert is a cartridge-based amplification test of nucleic acid that is used for the rapid diagnosis of tuberculosis).

### Data analysis

We performed a descriptive analysis of all the variables. The median and interquartile range (25th and 75th percentiles) were calculated in the following stages: 1) time in days from the time of the onset of respiratory symptoms to the diagnosis of TB; 2) time in days from the diagnosis of TB to the start of TB treatment; and 3) the total time in days from the onset of symptoms to the start of TB treatment. To explore which variables were associated with the time from of the onset of symptoms to TB diagnosis, a generalized linear model with a gamma distribution was used with an identity link function (11). For the variables included in the model, coefficients and their 95% confidence intervals were calculated using Stata version 11.0 software.

### Ethical aspects

The study was approved by the Ethics Committee of the Universidad Pontificia Bolivariana (Medellín, Antioquia, Colombia) and the Ethics Review Committee of the Pan American Health Organization (Washington, D.C., United States of America). Written approval was also obtained from the Ministry of Public Health of the city of Cali.

## RESULTS

In 2016, 90 public and private health care institutions from all levels of care reported a total of 983 cases to the Cali tuberculosis program. Based on the eligibility criteria, 623 patients with pulmonary TB were included in the study. Among these 623 persons, 57.0% were male, the median age was 42 years (interquartile range (IQR): 27–60), 70.0% were between 15 and 60 years old, and 51.0% belonged to the subsidized health insurance program (Table 1).

One-third of the patients had sputum smear-negative TB (n = 195) (Table 2). Among the total number of sputum smear-negative TB individuals, 92 (47.0%) were reported as positive based on culture and 12 positive based on GeneXpert results (data not shown).

When analyzing the time between onset of symptoms and TB diagnosis, the median time found was 57 days (IQR: 21–117); it was longer in women than in men, in mestizos as compared to indigenous persons, and in people between 30 to 44 years old as compared to those older than 75 years. Regarding the total number of days between TB diagnosis and initiation of treatment, the median was 7 days (IQR: 2–17); it was longer in women than in men. The median total time from the onset of symptoms to the initiation of TB treatment was 72 days (Table 1).

Despite the fact that all the population groups had a delay in the diagnosis of TB (more than 30 days), the time periods were longer in the following groups: female, mixed race, 30 to 44 years old, subsidized health insurance, drug dependent, and malnourished (Table 1).

**TABLE 1:** Sociodemographic characteristics and time from the onset of symptoms to diagnosis, from diagnosis to treatment initiation, and from onset of symptoms to treatment initiation in patients with pulmonary tuberculosis in Cali, Colombia, 2016 (N = 623)

Characteristic		N/n	%	Days (median) between onset of symptoms and diagnosis (Q1, Q3)^a^	Days (median) between diagnosis and initiation of treatment (Q1, Q3)	Total days (median) between onset of symptoms and treatment (Q1, Q3)
Total		623	100	57 (21–117)	7 (2–17)	72 (35–145)
Sex						
	Female	267	43.0	60 (26–103)	7 (2–23)	75 (40–125)
	Male	359	57.0	51 (18–141)	6 (2–13)	68 (31–157)
Ethnic group						
	Mestizo	524	84.0	59 (21–120)	6 (2–17)	73 (34–148)
	Afro-Colombian	92	15.0	50 (22–109)	8 (4–16)	73 (37–123)
	Indigenous	7	1.0	36 (22–88)	5 (0–7)	41 (22–95)
Age group in years						
	≤ 14	25	4.0	45 (12–99)	11 (5–72)	91 (35–122)
	15–29	171	27.5	50 (18–108)	5 (2–12)	65 (30–122)
	30–44	136	21.8	67 (31–154)	5 (2–12)	80 (38–173)
	45–59	123	20.0	55 (22–125)	7 (2–17)	64 (34–147)
	60–74	116	19.0	58 (19–1110)	10 (3–39)	72 (36–150)
	≥ 75	52	8.0	35 (16–108)	7 (3–27)	66 (30–132)
Population group						
	People incarcerated	37	6.0	35 (22–90)	4 (2–6)	41 (26–104)
	Pregnant women	3	0.4	72 (13–86)	4 (1–11)	83 (14–90)
	Displaced persons	12	2.0	69 (36–89)	8 (6–10)	84 (65–99)
	Persons with a disability	8	1.0	69 (20–150)	13 (1–33)	88 (30–190)
	Homeless persons	26	4.0	70 (23–358)	5 (0–12)	77 (31–358)
	Health care workers	12	2.0	30 (8–116)	10 (3–28)	81 (31–139)
	None of the above	525	84.0	58 (21–117)	7 (2–18)	74 (36–145)
Health insurance^b^						
	Contributory	249	40.0	43 (17–118)	6 (2–23)	63 (30–155)
	Subsidized	318	51.0	62 (26–116)	7 (3–14)	74 (38–139)
	Without health insurance	56	9.0	57 (28–122)	6 (2–11)	69 (38–140)
Substance use						
	Drug dependence	76	12.0	81 (31–179)	5 (1–11)	91 (38–187)
	Smoking	10	2.0	80(17–411)	9 (2–24)	104 (23–494)
	Alcoholism	17	3.0	80 (55–156)	6 (2–14)	118 (62–162)
	No consumption	520	83.0	52 (21–103)	7 (2–18)	70 (34–127)
Clinical condition						
	HIV seropositive	46	7.4	36 (13–80)	4 (1–12)	48 (31–98)
	Diabetes	57	9.2	48 (21–87)	2 (2–2)	71 (38–104)
	Malnutrition	72	11.6	71 (26–146)	5(1–13)	80 (35–175)
	No clinical condition	448	72.0	58 (21–125)	7 (3–18)	75 (35–147)

***Source:*** Prepared by the authors, based on the study results.

a Q1 = 25^th^ percentile; Q3 = 75^th^ percentile.

b The subsidized health insurance regime represents the poorest and most vulnerable population, without the ability to pay for the insurance cost. The contributory health insurance regime is mandatory for people with an employment contract, retirees, and self-employed persons who are able to pay for the insurance cost.

**TABLE 2:** Clinical characteristics and time in days from the onset of symptoms to diagnosis, from diagnosis to treatment initiation, and from onset of symptoms to treatment initiation in patients with pulmonary tuberculosis in Cali, Colombia, 2016 (N = 623)

Characteristic		n	Days between onset of symptoms and diagnosis, median (Q1, Q3)^a^	Days between diagnosis and initiation of treatment, median (Q1, Q3)	Total days between onset of symptoms and treatment, median (Q1, Q3)
Admission condition					
	New patient	542	54 (21–107)	6 (2–28)	71 (34–126)
	Previously treated TB patient	81	89 (26–353)	7 (2–15)	105 (38–381)
Smear result^b^					
	Positive +	168	55 (22–100)	5 (2–9)	61 (28–105)
	Positive ++	122	58 (18–105)	4 (1–8)	65 (26–122)
	Positive +++	138	68 (31–160)	5 (2–10)	73 (35–168)
	Negative	195	48 (17–125)	26 (6–53)	94 (46–169)
Culture result					
	Positive	280	61 (23–127)	8 (3–32)	84 (43–153)
	Negative	140	63 (21–132)	7 (3–18)	75 (31–179)
	Not performed	203	45 (19–100)	5 (1–10)	53 (30–104)
GeneXpert test result					
	Detected	107	66 (27–142)	5 (2–13)	75 (38–64)
	Not detected	18	24 (8–43)	14 (5–43)	54 (32–71)
	Not interpretable	1	67 (67–67)	23 (23–23)	90 (90–90)
	Not performed	273	46 (18–103)	6 (2–16)	61 (30–136)
	Not applicable	224	62 (24–122)	7 (3–20)	82 (39–142)

***Source:*** Prepared by the authors, based on the study results.

a Q1 = 25^th^ percentile; Q3 = 75^th^ percentile.

b The smear result refers to the number of bacilli identified by microscope: positive +: less than one bacillus per field is observed on average in 100 observed fields; positive ++: 1 to 10 bacilli are observed per field on average in 50 observed fields; positive +++: more than 10 bacilli are observed per field on average in 20 observed fields.

**TABLE 3:** Risk factors associated with the time in days from the onset of symptoms to TB diagnosis and TB treatment initiation in patients with pulmonary tuberculosis in Cali, Colombia, 2016

Variable		*P* value	Total time in days coefficient (95% CI)^a^	TB treatment initiation coefficient (95% CI)^b^
Constant		< 0.001	111.6 (89.90 to 133.4)	14.0 (9.11 to 18.90)
Contributive health insurance plan		0.140	21.9 (−7.5 to 51.3)	1.0 (−5.29 to 7.43)
Person incarcerated		0.001	−57.3 (−92.4 to −22.26)	−7.3 (−13.9 to −7.31)
Drug dependence		0.320	24.1 (−23.6 to 72)	−3.1 (−10.54 to 4.21)
Health care worker		0.200	−43.3 (−110.8 to 24)	−3.3 (−23.60 to 16)
Diabetes		0.080	−28.4 (−60.7 to 3.7)	−3.2 (−11.15 to 4.57)
Sex (female)		0.720	−4.9 (−32.5 to 22.6)	5.6 (−0.33 to 11.58)
Previously treated TB patient		0.001	123.8 (48.3 to 199.3)	14.0 (2.61 to 25.41)
Health care service institution rating^c^				
	70%–80%	0.080	52.5 (−7.5 to 112.5)	14.8 (0.20 to 29.50)
	Less than 70%	0.670	68.3 (−252.5 to 389.3)	3.0 (−41.66 to 47.77)

***Source:*** Prepared by the authors, based on the study results.

a Total time in days from the onset of respiratory symptoms to TB treatment initiation, with 95% confidence interval (CI).

b Time in days from TB diagnosis to TB treatment initiation.

c The health care service institution rating is the percentage of compliance with regulations for tuberculosis, according to the rating technical assistance instrument.

More days elapsed from the onset of symptoms until the diagnosis in those patients who had been previously treated and in those with a negative culture (Table 2).

A factor associated with longer time from the onset of symptoms to TB treatment was being a previously treated TB patient. Being incarcerated was a protective factor for earlier TB treatment initiation (Table 3).

## DISCUSSION

This report is among the first attempts to determine the risk factors associated with delay in TB diagnosis and TB treatment initiation in pulmonary TB patients in the city of Cali. Our results show that despite all the global and national strategies and guidelines implemented by the Colombian TB program to identify people with respiratory symptoms, the median time between the onset of symptoms and TB treatment initiation in Cali was 72 days. Additionally, of the total number of patients included in the study (N = 623), 75.0% had ≥ 30 days between the onset of symptoms and treatment initiation. These findings highlight the important challenges that local TB programs have in achieving their goals, given that the maximum time between symptoms and treatment should 32 days (maximum of 30 days for TB diagnosis and 2 days for TB treatment initiation) (3, 13).

Research conducted in 2015 in Lima, Peru, to determine factors associated with diagnostic delay (14) found results similar to those in our study. In both Lima and Cali, the median time between onset of symptoms and the diagnosis was 57 days. In addition, when comparing the data by sex, the findings in Lima were similar to those in Cali, with the median delay in Lima being 62 days for women and 50 days for men.

Some studies conducted elsewhere in the world have reported a lower number of days between symptoms and TB treatment than what we found in Cali. This is the case for Brazil, with a median of 60 days (13); Ethiopia, with 70.5 days (8); and eight cities of Colombia, with a median of 51 days (9). On the other hand, some other studies have reported median delays of as much as 120 days (6, 15, 16).

The delay in the initiation of TB treatment in our Cali study is alarming, considering that this can lead to an increase in the spread of TB in the community. A total of 69% of the patients in Cali had a sputum smear-positive result, with 42% of the total with either a “++” or “+++” result (Table 2).

Of the Cali cases for which a molecular test such as GeneXpert was indicated, it was only performed in 65.0% of those patients. This finding shows the need to identify the possible barriers to accessing this molecular test, which can detect both TB and resistance to rifampicin (18, 19).

Medical care personnel can play a fundamental role in the late diagnosis of pulmonary tuberculosis. For example, beliefs associated with social stigma and insufficient knowledge about the disease—shared by the medical staff and patients—can contribute to the social construction of the stereotype of the disease and the patient. Those circumstances can reduce the chances of reaching an opportune diagnosis of pulmonary tuberculosis (20).

Even though we did not collect information on beliefs and knowledge of health staff in our Cali study, attendance at a health service institution that was rated between 70% and 80% on compliance with national TB program guidelines was a factor associated with delay in the initiation of tuberculosis treatment. This indicates the need for the TB program in Cali to reinforce adherence to national TB protocols and norms as well as to evaluate strategies and public policies related to TB diagnosis and early initiation of TB treatment.

Our data shows that previously treated patients had a greater delay in starting TB treatment—up to 123 days—as compared to recently admitted patients. A study conducted in India showed that the delay in TB diagnosis was 1.8 times higher among previously treated TB patients than in new TB cases (20), which is in line with our results. These differences in findings among the studies may be related to variations in the study populations, study settings, and socioeconomic backgrounds in the respective studies.

In our study, being incarcerated decreased the time of initiation to TB treatment up to 57 days in comparison with the general population. This could be explained by the reinforcement of TB control in prison in Cali and the characteristic of the TB program in this type of setting, where health care promoters and inmates affected by the disease have been trained to carry out active case findings of people with respiratory symptoms. It has been demonstrated that peer educators are effective participants in this process (21). Governmental commitment, partnerships, and sustained financing will be needed in order to continue these interventions in Cali.

Some factors should be taken into account when interpreting the results of this study. This was an operational study and therefore subject to the limitations of such research. We could only assess variables that are routinely collected in the registers, and there may be other factors related to delay that we were not able to assess. Furthermore, from the register reviews. we could not differentiate between patient-related delays and provider-related delays.

There were a number of noteworthy results in our study. One was the delay in diagnosis, with a median of 57 days. Another was that 25.0% of people will start treatment within 17 days after being diagnosed. A third was that the delay in diagnosis was greater in women, in people in the subsidized insurance regimen, and in people with some degree of malnutrition. A fourth was that were several factors associated with the delay in the diagnosis and the beginning of the treatment.

There are four important needed actions that this study has identified for the control of tuberculosis in the city of Cali. The first is to evaluate and update local TB program strategies and public policies related to TB diagnosis and early initiation of treatment. The second is to improve educational and health promotion activities in the Cali TB program, focusing them on patient delays. The third is to improve the efficiency of health care facilities by updating Cali's TB protocols, strengthening adherence to these reference documents, and training health care personnel. The fourth is to strengthen the links between health care institutions and the community.

## Author contributions.

Córdoba designed the study, undertook data collection and data analysis, and wrote the paper. Luna and Triana contributed to writing the manuscript. Perez participated in data analysis, manuscript preparation, and overall editing. López was involved with study design, data analysis, and manuscript preparation.
